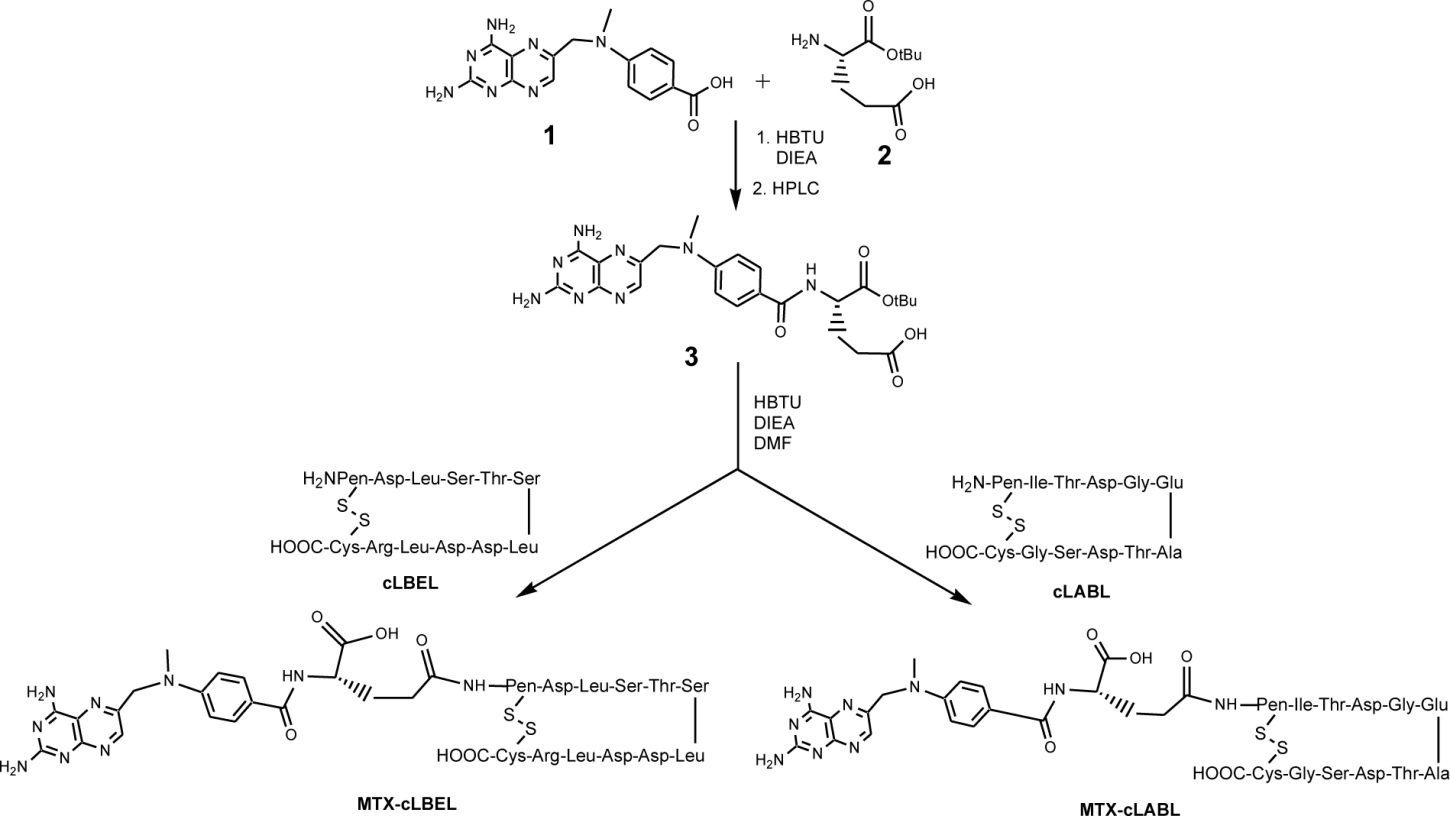# Evaluation of LFA-1 Peptide-Methotrexate Conjugates in Modulating Endothelial Cell Inflammation and Cytokine Regulation

**DOI:** 10.18103/mra.v11i2.3534

**Published:** 2023-02

**Authors:** Helena Yusuf-Makagiansar, Tatyana V. Yakovleva, Meagan Weldele, Rucha Mahadik, Teruna J. Siahaan

**Affiliations:** 1 Department of Pharmaceutical Chemistry, The University of Kansas, 2095 Constant Avenue, Lawrence, KS 66047; 2 Mapp Biopharmaceutical Inc., 6160 Lusk Blvd. # C105, San Diego, CA 92121; 3 Department of Medicinal Chemistry, College of Pharmacy, University of Florida, 1225 Center Drive, Gainesville, FL 32610; 4 Johnson County Community College, 12345 College Blvd, Overland Park, KS 66210

**Keywords:** LFA-1 peptides, Peptide-drug conjugates, Methotrexate, Targeting ICAM-1 receptor, Targeted Delivery, Inflammatory Suppression, coronary atherosclerosis

## Abstract

Interactions between vascular endothelial cells and inflammatory leukocytes are intermediated via cell adhesion molecules and they become one of the key events for vascular cell injury and development of atherosclerosis. This study evaluated the effects of MTX-peptide conjugates as anti-inflammatory agents on human coronary artery endothelial cells (HCAEC) and Molt-3 T cells. Cyclic peptides, cLABL and cLBEL, were derived from the α- and β-subunits of leukocyte function-associated antigen-1 (LFA-1), respectively. They interact with intercellular adhesion molecule-1 (ICAM-1) to inhibit LFA-1/ICAM-1-mediated homotypic or heterotypic T-cell adhesion. cLABL and cLBEL were linked to the anti-inflammatory drug, methotrexate (MTX), to produce MTX-cLABL and MTX-cLBEL conjugates. This study showed that peptides and MTX-peptide conjugates inhibited T cell adhesion to HCAEC monolayers while MTX alone did not. The conjugates, but not MTX, inhibited binding of anti-ICAM-1 monoclonal antibody (mAb) to ICAM-1 on the HCAEC. This indicates that conjugation of MTX to cLABL and cLBEL peptides did not dramatically change their binding properties to ICAM-1. The conjugates had relatively lower toxicity to cells compared to MTX alone, while they were more toxic than the parent peptides. At low concentrations, MTX, MTX-cLABL and MTX-cLBEL decreased production of IL-6 and IL-8 as inflammatory cytokines. In contrast, higher concentrations of the parent peptides compared to the conjugates were required to inhibit IL-6 and IL-8 productions. Overall, both MTX-cLABL and MTX-cLBEL were more active than both free-peptides. In addition, the conjugates were less toxic than MTX alone. In conclusion, the conjugate can selectively target MTX to ICAM-1-expressing cells to increase cell targeting and to lower MTX toxicity.

## INTRODUCTION

Leukocyte extravasation and migration to inflamed tissues have been associated with many pathophysiological conditions, including coronary atherosclerosis, endothelial cell injury, and myocardial infarction.^1^ The sequential steps during leukocyte-endothelial contacts involve a set of adhesion molecules. One of the critical markers of inflammatory response is the intercellular cell adhesion molecule-1 (ICAM-1).^2–4^ ICAM-1 is expressed on most cells with leukocyte function-associated antigen-1 (LFA-1) as the main receptor on T cells. ICAM-1 has been the target for monoclonal antibody (mAb) inhibition to suppress inflammation in several models of endothelial inflammation.^5–7^ There is a specific interest to develop small molecule inhibitors for anti-adhesion therapy with sustained ligand-binding activity and immunogenicity. For this purpose, adhesion peptides may serve as an alternative to mAbs. Cell adhesion peptides and peptidomimetics derived from the Arg-Gly-Asp (RGD) sequence have emerged as antithrombic agents (i.e., Integrilin, Agrastat^®^) as well as antitumor and cancer diagnostic agents.^8–12^ Thus, cell adhesion peptides have become an attractive type of molecules as therapeutic or diagnostic agents that bind to cell adhesion receptors on the surface of disease cells.

Cell adhesion peptides that intervene with ICAM-1/LFA-1 interactions rely on their sequence and conformation for their receptor binding properties. Interaction of ICAM-1 and LFA-1 is mediated by binding between the D1 domain of ICAM-1 and the insert (I)-domain of LFA-1.^12–17^ The I-domain comprises of about 200 amino acid residues at the N-terminal of the LFA-1 α-subunit. Domains V and VI^18^ as cation binding domains and the β_2_ subunit I-like domain^19,20^ are other binding regions of LFA-1. Peptides with sequences derived from the I-domain of LFA-1^1,21–24^ along with peptides derived from the sequence of the ICAM-1 domain^25–28^ can inhibit cell adhesion. An LABL peptide derived from the α-subunit of LFA-1 blocked ICAM-1/LFA-1 interaction through ICAM-1 binding.^29,30^ In addition to cell adhesion, ICAM-1/LFA-1 interactions have a role in differentiation and proliferation of immune cells because they act as a co-stimulatory signal during the activation of T cells.^31–34^ In autoimmune diseases, stimulation of proinflammatory T cells involves ICAM-1/LFA-1 interactions in propagating autoimmune response. Previous work has implied that LABL-antigenic peptide conjugates can alter autoimmune inflammatory response to an antigen-specific tolerogenic responses.^31,35–38^

Our previous work had shown that cLABL peptide (cyclo1,12-PenITDGEATDSGC) derived from the LFA-1 I-domain interacted with the ICAM-1 D1 domain^23,39^ to inhibit adhesion of lymphocytes^29,30^ in an *in vitro* model of epithelial cell inflammation.^22^ This peptide was synthesized by incorporating cysteine (Cys) and penicillamine (Pen) residues to the C- and N-termini, respectively, on the Ile^237^–Gly^246^ sequence of the I-domain. Then, the oxidation of the thiol groups of the Pen and Cys residues formed a disulfide bond, generating the cyclic structure.^21^ The restricted conformation of cyclic peptide could increase plasma stability and receptor binding selectivity to the targeted receptor compared to the parent linear peptide. Compared to phosphate buffer saline (PBS) as a negative control, cLABL peptide significantly suppressed arthritis symptoms in collagen-induced rheumatoid arthritis (CIA) mouse model similar to treatments with anti-CD11a and anti-ICAM-1 mAbs as positive controls.^40^

The cyclic peptide cLBEL (cyclo1,12-PenDLSTSLDDLRC) inhibited T cell adhesion to monolayer of epithelial cells via binding to ICAM-1.^29,30^ This peptide has a sequence from the I-like domain of LFA-1 β-subunit, which has approximately 250 amino acids located at the N terminus.^29,30^ The I-like domain has the most conserved sequence in the integrin family.^19,20^

In this study, cLABL and cLBEL peptides were linked to methotrexate (MTX) to produce MTX-cLABL and MTX-cLBEL conjugates. The goal was to target MTX to human coronary artery endothelial cells (HCAEC) via the ICAM-1 receptor to lower MTX toxicity and side effects. The biological abilities of MTX-cLABL, MTX-cLBEL, cLABL, cLBEL, and MTX were compared by their activities to inhibit binding of anti-ICAM-1 mAb to ICAM-1 on the surface of HCAEC. In addition, these molecules were compared in inhibiting T cell adhesion to HCAEC monolayers. Finally, their activities in suppressing IL-6 and IL-8 production as inflammatory cytokines were determined. The toxicities of MTX-cLABL and MTX-cLBEL conjugates were also determined relative to MTX alone as well as cLABL and cLBEL peptides.

## MATERIALS AND METHODS

### Cell Cultures:

Molt-3 T cells were obtained from the American Type Culture Collection (Rockville, MD). They were maintained using RPMI1640 media containing 100 μg/l of penicillin/streptomycin along with 10% heat-inactivated fetal bovine serum (FBS). Tissue culture flasks (75-cm^2^; Falcon, Fisher, Pittsburgh, PA) were used to culture T cells at 37°C under a saturating humidified atmosphere of 95% air and 5% CO_2._ Phorbol 12-myristate-13-acetate or PMA (0.2 μM; Sigma, St. Louis, MO) was used to activate these cells. HCAEC (Clonetics, San Diego, CA) were propagated and maintained using medium provided by the vendor in tissue culture flasks (75-cm^2^; Falcon, Fisher, Pittsburgh, PA). HCAEC were activated with 20 ng/ml of TNF-α (Millipore Sigma) for 24 h.

### Synthesis of Methotrexate-Peptide Conjugates:

The solid phase peptide synthesizer was used to make linear precursors of cLABL and cLBEL cyclic peptides. The crude products were subjected to purification with a semi-preparative C18 column on HPLC. An air oxidation of the linear peptide precursor in high dilution solution was carried out to generate the cyclic peptide. The cyclic peptide was further purified by HPLC and analyzed by fast atom bombardment mass spectrometry (FABMS). The MTX γ-carboxylic acid was conjugated to the N-terminus of cLABL or cLBEL peptide via a peptide bond to make MTX-cLABL or MTX-cLBEL conjugate, respectively, as described previously (Scheme 1).^41^ Compound **1** carboxylic acid was activated using benzotriazolyloxytetramethylivonium hexafluorophosphate (HBTU) and N,N-diisopropylethyl amine (DIEA) in N,N-dimethyl formamide (DMF). The product was then conjugated to the amine group in compound **2** (Glu(O-tBu)-OH) to generate compound **3** with protected α-carboxylic acid at 85–92% yield after purification by semi-preparative HPLC. The Glu γ-carboxylic acid in compound **3** was activated by HBTU in the presence of DIEA in DMF followed by addition of cLABL peptide to produce MTX-cLABL with tert-butyl (t-Bu) protected group. Then, the t-Bu protecting group in the α-carboxylic acid of Glu amino acid was removed by a 1:1 ratio of TFA in methylene chloride (CH_2_Cl_2_) and stirred for 45 min. A semi-preparative HPLC with C18 column was used to purify the crude product. The pure MTX-cLABL showed the correct molecular weight by FABMS (M+1=1634 amu). A similar route was used to produce MTX-cLBEL with the correct molecular weight (FABMS; M+1 = 1428 amu).

### Flowcytometry:

The ICAM-1 expression on TNF-α-activated HCAEC cells were determined by flowcytometry using detached cells; the detached cells were produced upon exposure of cell monolayers by EDTA (0.01 %) in PBS for 10 min at 37°C that subsequently subjected to gentle trituration. The cells were subjected to centrifugation to generate pelleted cells; then, 4°C in 100 μl solution of FITC-labeled anti-ICAM-1 mAb (clone BBIG-I1, Chemicon, Temecula, CA) with a final concentration of 10 μg/ml. The mAb binding to cell surface was quantified using a fluorescence-activated cell sorter (FACScan, Becton Dickinson; Cell Quest Software) at a total of 5×10^3^ cells (100% gated). Irrelevant mouse IgG_1_ (Sigma) with nonspecific binding property was used as a negative control. FACScan was used to analyze the inhibitory activity of test compounds in inhibiting mAb binding to ICAM-1. The test compound at 1.0–100 μM was added to HCAEC cultures in 12-well cell culture plates. After incubated with test compound at 4°C for 4-h, the cells were incubated with FITC-labeled anti-ICAM-1 mAb to determine their activities to inhibit mAb binding.

### Heterotypic Cell Adhesion Assay:

The heterotypic T cell adhesion was determined using previously described method.^22^ Briefly, HCAEC monolayers was activated with TNF-α followed by treatment with test compounds or anti-ICAM-1 mAb (clone 11C81, R&D Systems, Minneapolis, MN) before the addition of Molt-3 T cells with fluorescence label. PMA was added into Molt-3 T cells suspension followed by incubation for 24 h for cell activation. Then, the T cells (3 × 10^7^/ml) were incubated for 1 h with 50 μg BCECF (Molecular Probes, OR) in 50 μl dimethyl sulfoxide to label the cell cytoplasmic domain. The free label was removed by washing 10^6^/ml cells with serum-free RPMI1640 medium. Peptides in RPMI-HEPES (12.5–100 μM) were added to HCAEC monolayers followed by incubation at 4°C for 30 min. After washing with RPMI-HEPES, HCAEC monolayers were incubated with labeled Molt-3 T cells at 37°C for 45 min. The cell adhesion mixture was washed with HEPES/PBS three times. After washing, the cells were lysed with 0.5 ml of 2% Triton X-100 in PBS. The cell lysates were transferred to 96-well plates (clear-bottom, black-sided; Costar) to determine the fluorescence intensity (FL) from the Molt-3 T cells using a microplate fluorescence analyzer (Bio-Tek FL600). The fluorescence intensity of cell monolayer was used to correct the contribution from HCAEC. Percentage of T cell adhesion was calculated using the following equation:


Percentofcelladhesion(%)=(FLoftreatedsamples/FLofcontrol)×100

### ELISA:

TNF-α (20 ng/ml) was added to HCAEC monolayers in 48-well plate; then, the cells were incubated with and without test compounds for 24-h at 37°C under 5% CO_2_ atmosphere. Then, concentrations of IL-6 and IL-8 cytokines in supernatants were quantified using a competitive enzyme immunoassay (EIA; Chemicon).

### Cytotoxicity Assays:

Propidium iodide (PI) assay was used to determine the levels of double stranded polynucleic acid (PNA) as an indication of cell viability using previously published method.^42^ HCAEC cells were cultured in 96-well cell plate in GIBCO non-CO_2_-buffered culture medium (Life Technologies, Gaithersburg, MD) supplemented with 2.0 mM L-glutamine (Sigma) and 5% fetal calf serum. Determination of compound cytotoxicity, the assay utilizes a 1 + 2-day screening protocol from the U.S. National Cancer Institute (NCI). In this case, 1 day recovery was implemented for the cells after the dissociation trauma. Then, the test compound was added into the cells and incubated for 2 days. After the incubation, cells were frozen for 2 h at – 30°C followed by thawing at 50°C for 15 min. Each well was added with 40 μg/ml of PI followed by incubation in the dark at room temperature for 60 min. Then, the fluorescence intensity of PI was determined with excitation at 530-nm and emission at 620-nm. The final fluorescence intensity was determined by subtracting fluorescence contributions from the test compound, cells, and culture medium. The amount of cellular PNA was used to determine the effect of test compound on cell growth and cytotoxicity. The magnitude of toxicity was categorized into different levels such as total culture extinction, net cell killing, total growth inhibition, partial growth inhibition, and growth stimulation.^42^

### Fluorescence Microscopy:

The cell adhesion of fluorescence-labeled T cells to activated HCAEC monolayer was observed using Nikon fluorescence microscope with Flash Point software. Representative data from 100× magnifications were presented in black and white images.

### Statistical Analysis:

The data presented as the mean ± SE. A Student’s t-test was used to determine the statistical significance between groups. A statistical significant difference was established at *P*<0.05.

## RESULTS

### Heterotypic T-cell Adhesion and Anti-ICAM-1 mAb Inhibition:

**Figure 1.**
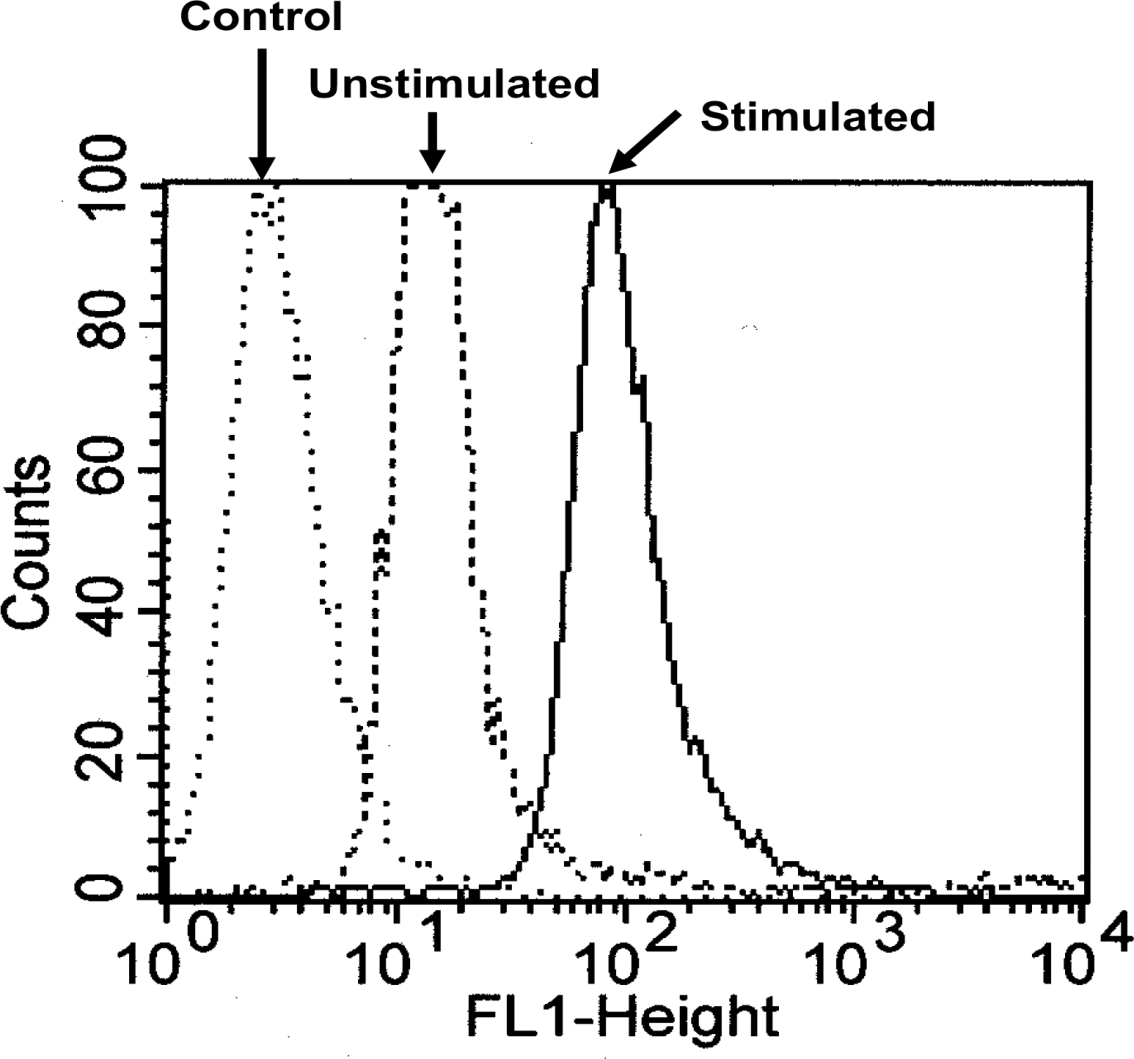
The ICAM-1 expression on HCAEC was determined using anti-ICAM-1 mAb by flow cytometry. Cell monolayers were activated for 24 h by TNF-α (20 ng/ml) and the single cells have increased ICAM-1 expression (Stimulated) compared to a basal expression of ICAM-1 on nonactivated HCAEC (Unstimulated). The negative control cells were not incubated with anti-ICAM-1 mAb with a very low fluorescence intensity.

**Figure 2.**
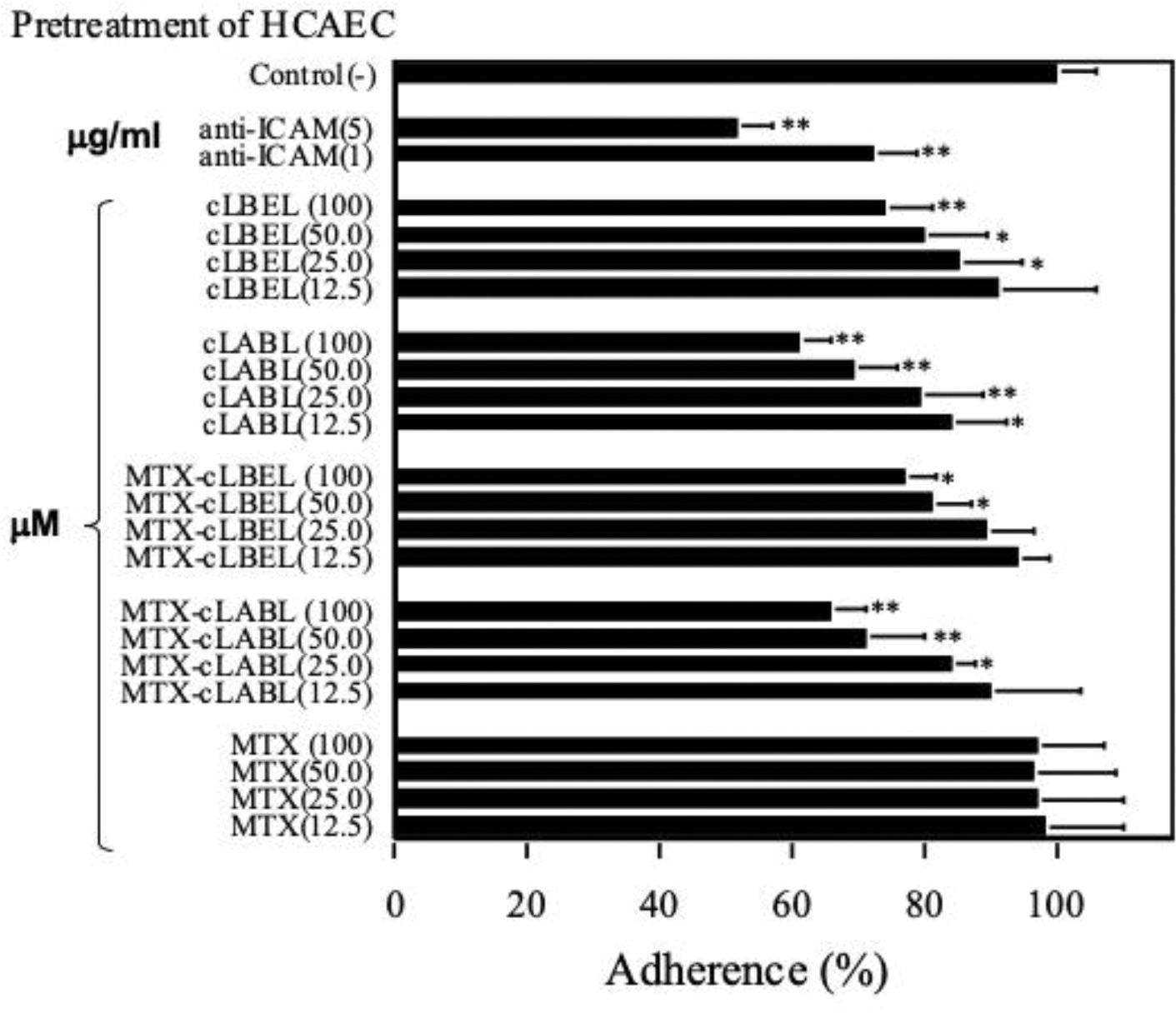
Inhibition of heterotypic T-cell adhesion to endothelial cells by peptides, MTX, MTX-peptide conjugates and anti-ICAM-1 antibody. Confluent stimulated HCAEC were treated with the test compound at various concentrations, as indicated in parentheses, before the addition of fluorescent labeled activated Molt-3 cells. All test compound concentrations were in μM. The antibody was delivered at 1.0 and 5.0 μg/ml. *P<0.05 and **P<0.01 as compared with control in which T-cell adherence to HCAEC monolayers treated with irrelevant peptide.

The inhibitory activities of cLABL, cLBEL, MTX-cLABL, MTX-cLBEL, and MTX were evaluated in reducing T cell adhesion to HCAEC monolayer. Cell activation was performed prior to the cell adhesion experiment by inducing both HCAEC cell monolayers and Molt-3 cells with TNF-α and phorbol ester (PMA), respectively. ICAM-1 is constitutively expressed in HCAEC and can be up regulated by TNF-α activation (Figure 1). The peptides and conjugates inhibited T cell adhesion in a concentration-dependent manner (Figure 2). In contrast, MTX was completely inactive in this heterotypic adhesion assay. The activity rank of compunds to inhibit T cell adhesion at 100 μM was as follows: cLABL > MTX-cLABL > cLBEL > MTX-cLBE. The inhibitory activity of conjugate decreased compared to parent peptide by 6 to 10%.

**Figure 3.**
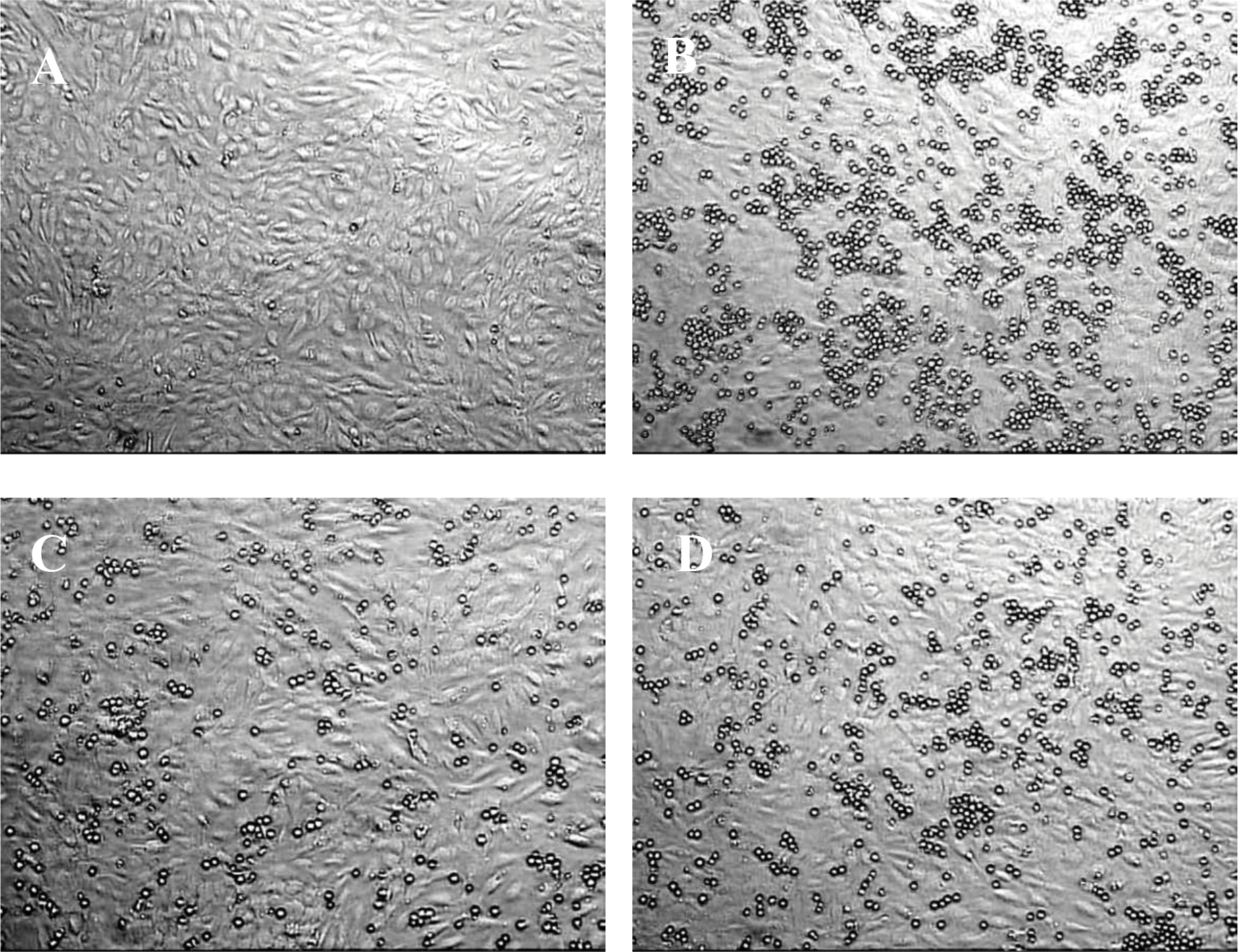
Molt-3 T cell adhesion to HCAEC monolayer was observed by microscopy analysis. **(A)** Representative microscope images of HCAEC monolayers only. Molt-3 T cell adhesion to HCAEC monolayer was inhibited by the following agents: **(B)** control peptide; **(C)** anti-ICAM-1 mAb; and **(D)** MTX-cLABL conjugate.

The effect of MTX-cLABL of T cell adhesion to HCAEC monolayer was visualized in Figure 3 with the control for HCAEC monolayer in Figure 3A. A higher T cell adhesion was observed when treated with a control molecule (Figure 3B) compared to that of treated with anti-ICAM-1 mAb (Figure 3C) or MTX-cLABL (Figure 3D). Anti-ICAM-1 mAb at 5.0 μg/ml (Figure 3C) was more effective in reducing T cell adhesion than 100 μM of MTX-cLABL (Figure 3D).

**Figure 4.**
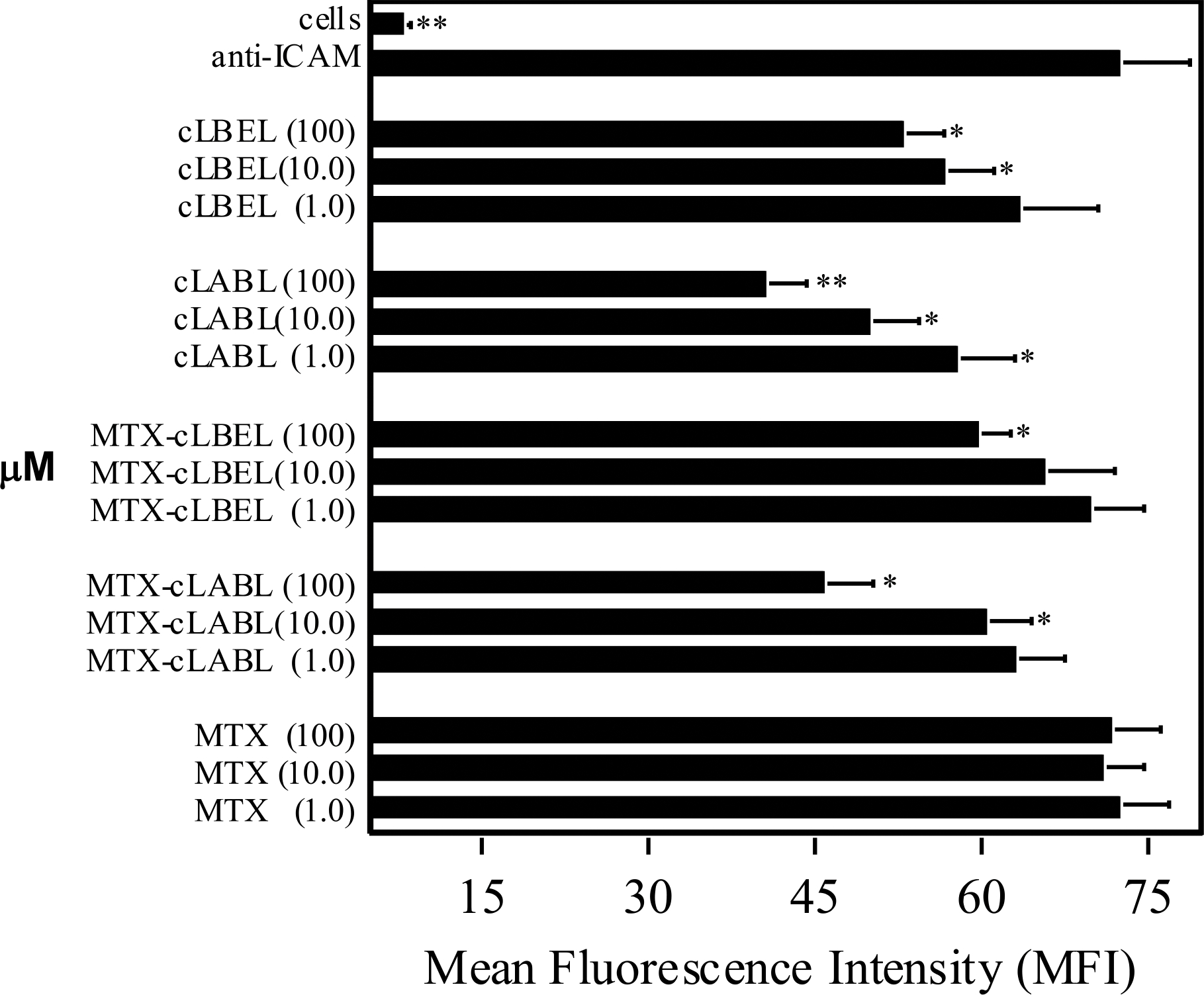
Inhibition of anti-ICAM-1 mAb binding to ICAM-1 on HCAEC monolayer by peptides, MTX-peptide conjugates, and MTX. Stimulated HCAEC were treated with the compounds at various concentrations (μM), as indicated in parentheses, before the addition of fluorescent-labeled anti-ICAM-1 mAb. *P<0.05 and **P<0.01 as compared with control which is binding of anti-ICAM-1 mAb on HCAEC treated with irrelevant peptide.

The heterotypic adhesion assays were confirmed with an anti-ICAM-1 mAb binding study (Figure 4). Stimulated single cells of HCAEC were treated with the test compound prior to immunostaining with anti-ICAM-1 mAb and analyzed using flow cytometry. As in the heterotypic T cell adhesion assay, all the test compounds, with exception of MTX, inhibited anti-ICAM-1 mAb binding to HCAEC in a concentration-dependent manner (Figure 4). Based on 100-μM concentration of test compounds, the activity to inhibit anti-ICAM-1 mAb binding has the following rank: cLABL (44.0%) > MTX-cLABL (36.7%) > cLBEL (27.0%) > MTX-cLBEL (17.5%). The conjugates showed a 7.3 to 14.4% reduction in inhibitory activity compared to the respective free peptides.

### Compound Relative Toxicities:

**Figure 5.**
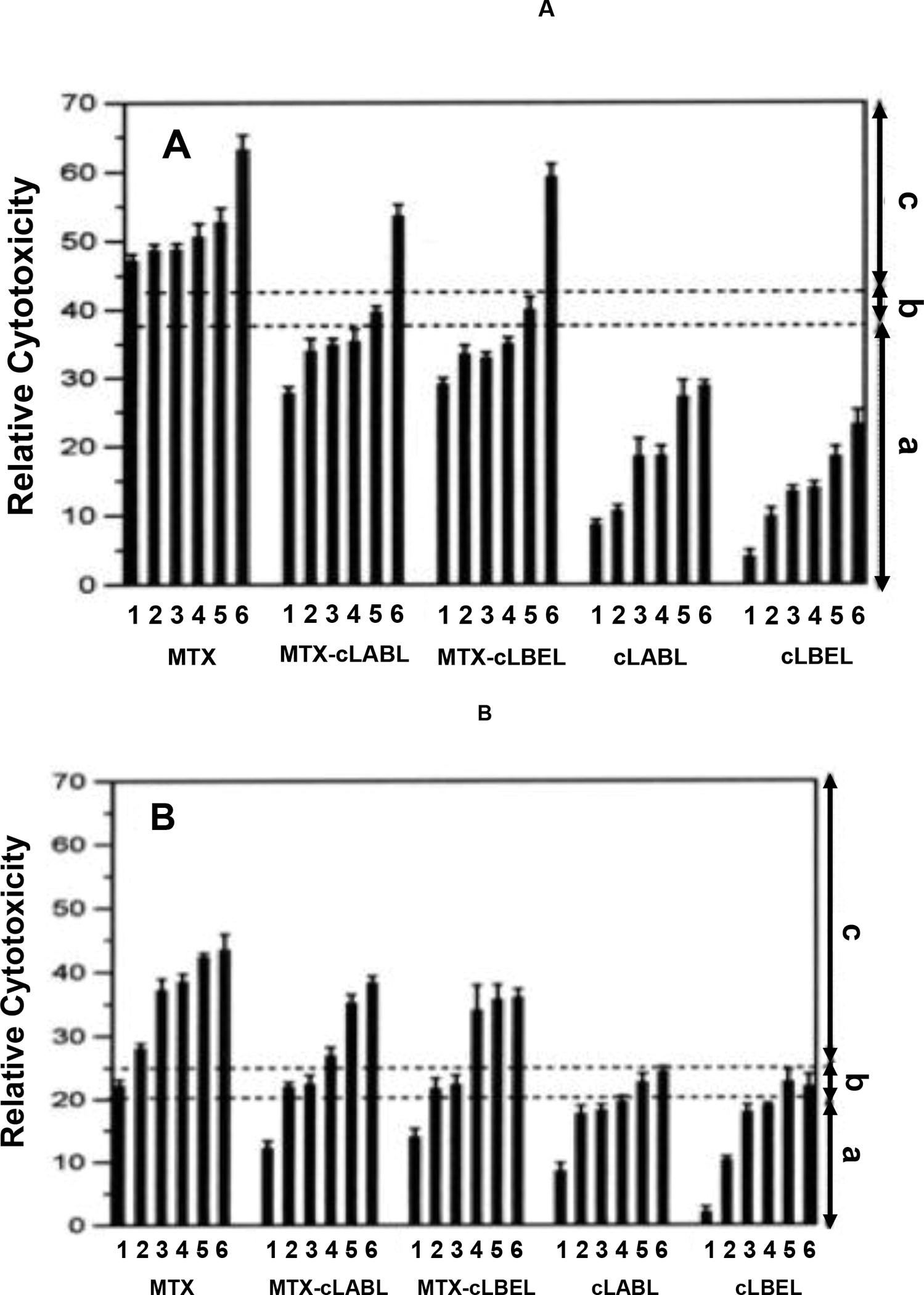
The effects of peptides, MTX-peptide conjugates, and MTX on cytotoxicity and growth of **(A)** HCAEC and **(B)** Molt-3 T-cells. Bar 1 to 6 are representing various compound concentrations of (1) 0.1 μM; (2) 1.0 μM; (3) 10 μM; (4) 50 μM; (5) 100 μM; and (6) 500 μM, respectively. The remaining amount of cellular poly-nucleic acids (PNA) was used to determine the effect of compounds on relative cytotoxicity levels such as **(a)** partial growth inhibition, **(b)** total growth inhibition or **(c)** net cell killing.

We next determined whether treatment of HCAEC and Molt-3 T cells with peptides, MTX, and MTX-peptide conjugates resulted in inhibition of cell proliferation. Both HCAEC and Molt-3 T cells were affected by test compound in different levels (Figures 5A and 5B). None of the molecules caused growth stimulation or total culture extinction. A net cell killing of HCAEC was observed upon treatment with MTX at all test concentrations (Figure 5A) while MTX affected net killing at ≥1.0 μM in Molt-3 T cells (Figure 5B). The MTX-peptide conjugates were less toxic than MTX. In HCAEC, the net cell killing was at lower concentration for MTX at ≥0.1 μM compared to MTX-peptide conjugates at ≥500 μM (Figure 5A). The net cell killing of Molt-3 T cells was found at ≥1.0 μM for MTX and ≥50 μM for MTX-peptide conjugates (Figure 5B). For all test concentrations, the conjugates only resulted in HCAEC partial growth inhibition (Figure 5A). For Molt-3 T cells, a total growth inhibition emerged at 100 μM for cLABL and cLBEL (Figure 5b); however, 500 μM cLABL and cLBEL did not cause total cell killing for T cells.

### Modulation of Inflammatory Cytokine Production:

**Figure 6.**
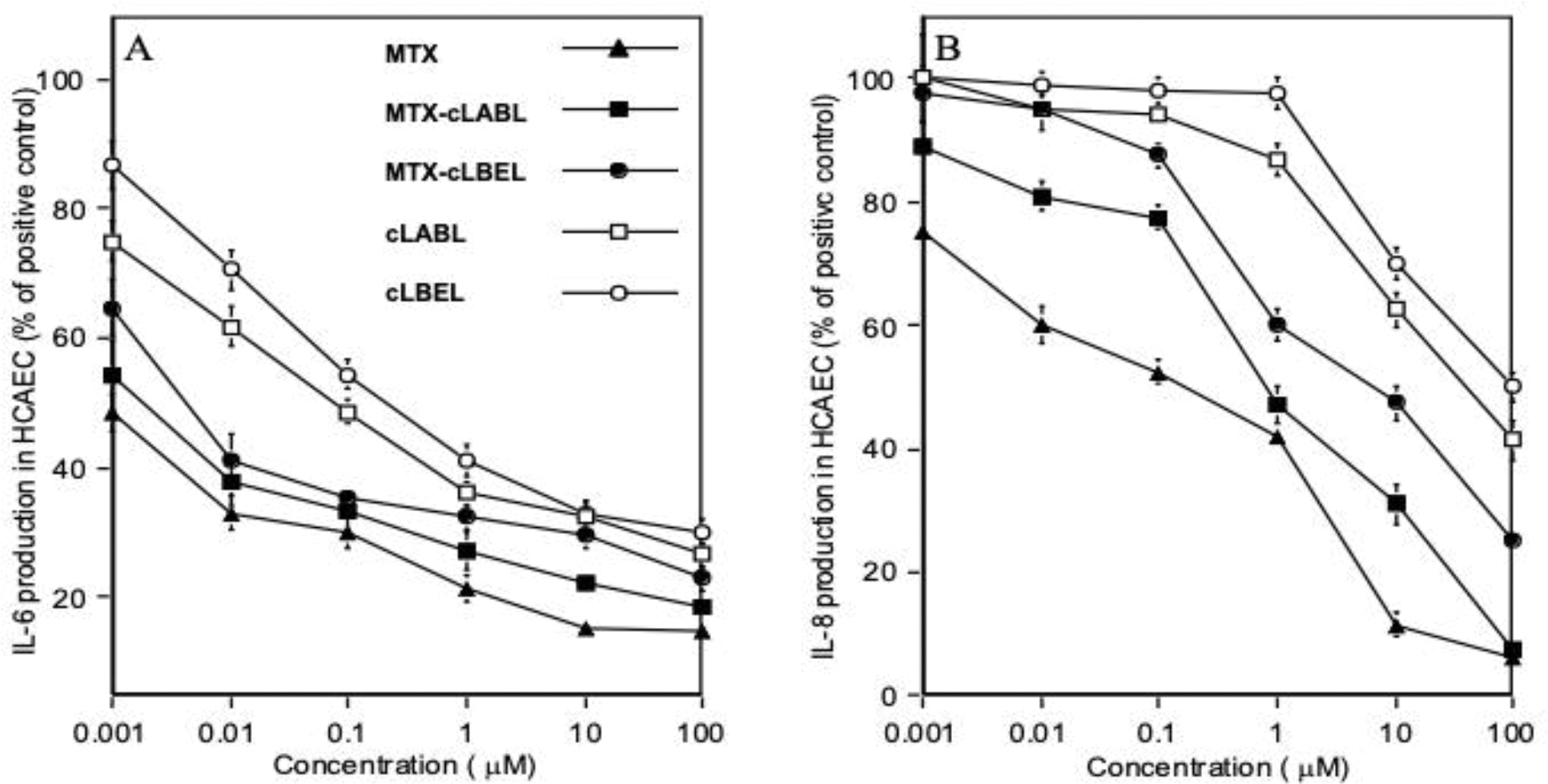
Effect of peptides, MTX-peptide conjugates, and MTX on the production of **(A)** IL-6 and **(B)** IL-8 from HCAEC. The test compounds (0.001 to 100 μM) were added to TNF-α-activated HCAEC and the results were expressed as the percentage of cytokine relative to the positive control. The non-treated monolayer was used as the baseline as 100% cytokine production. The control levels were expressed as mean ± SE with IL-6 = 3.4±0.17 ng/ml and IL-8 = 199.5±9.13 ng/ml.

The production of inflammatory cytokines could be modulated by anti-inflammatory agents. Here, peptides and MTX-peptide conjugates (0.001–100 μM) were evaluated to suppress IL-6 and IL-8 production in TNF-α-stimulated HCAEC monolayers (Figure 6). MTX and MTX-peptide conjugates have better activities than the respective free peptides in suppressing IL-6 and IL-8 production. MTX and MTX-peptide conjugates partly blocked IL-6 production with relatively comparable potency. Alternatively, the cLABL and cLBEL peptides required a 100-fold concentration increase in comparison to MTX-peptide conjugates to suppress larger than 50% of IL-6 production (Figure 6A). At ≥0.1 μM, MTX-peptide conjugates only begin to effectively reduce the production of IL-8 production; in contrast, neither cLBEL nor cLABL affected the production IL-8 at ≤1.0 μM (Figure 6B). In conclusion, peptide conjugation decreased the activity of MTX in controlling cytokine production compared to MTX; both conjugates and MTX affected suppression of IL-6 production more effectively compared to IL-8.

## DISCUSSION

Growing evidence suggests that ICAM-1 expression is upregulated in various immunological disorders including tissues undergoing autoimmune injury and inflammation as well as in malignant tumors.^1,43^ In this report, MTX-cLABL and MTX-cLBEL maintained their ICAM-1 binding properties. The selection of MTX as a drug payload was due to its efficacy as an anti-inflammatory drug. Unfortunately, MTX has severe side effects such as suppression of bone marrow production.^44^ Although there are several proposed MTX mechanisms of action, its antifolate activity is the most commonly recognized mechanism.^45,46^ In this case, MTX works at inhibiting purine and pyrimidine synthesis to subsequently suppress immune cell activation and proliferation. It is proposed that the conjugate reduced the inflammatory response using two potential mechanisms of action. First, the conjugate binds to ICAM-1 and inhibits T cell adhesion to inflammatory endothelium to prevent T cell activation. Second, the conjugate undergoes ICAM-1-mediated internalization into HCAEC to suppress production of inflammatory cytokines by MTX.

Indeed, free peptides and the conjugates, but not MTX, blocked ICAM-1-binding to anti-ICAM-1 mAb at various concentrations, signifying that the peptides and conjugates bind to ICAM-1 (Figure 4). The inhibition of both heterotypic T cell adhesion and binding of anti-ICAM-1 mAb to HCAEC monolayers by cLABL and cLBEL agreed with our previous data of peptide inhibition of T cell adhesion to the monolayer of epithelial cells.^22,23^ It was notable that MTX alone failed to inhibit T cell adhesion to HCAEC monolayers (Figure 2) because MTX did not bind to ICAM-1. This also supported the inability of MTX to block ICAM-1 binding to anti-ICAM-1 mAb on HCAEC (Figure 4). Therefore, it is unlikely that the MTX fragment on MTX-peptide conjugate was involved in inhibiting the heterotypic T cell adhesion.

The T cell adhesion levels to HCAEC upon inhibition by peptides and conjugates were never below 50%; this is due to the involvement of multiple receptors during T cell adhesion to endothelium stimulated by TNF-α (Figure 2). In reference to the activity of 5.0 μg/ml of anti-ICAM-1 mAb, the peptides and conjugates caused a significant decrease of T cell adhesion (Figure 2). The overall result suggests that cLABL is a better inhibitor than cLBEL. The potent *in vitro* efficacy of cLABL is due to peptide sequence and conformation.^12,39^ Four residues such as Asp^239^,^47^ Glu^241^,^48^ and Thr^243^ and Ser^245^ have been identified as critical residues for ICAM-1 binding.^12,49^ In addition, the DGEA sequence in cLABL has a similar structure compared to the same sequence found in LFA-1, indicating its importance for binding to ICAM-1.^39^ On the other hand, Asp^134^ and Ser^136^ residues on cLBEL reside in the metal ion-dependent adhesion site (MIDAS) of β_2_-subunit in α_L_β_2_ (LFA-1) and α_M_β_2_ (Mac-1). They have been suggested to be important for the cation-dependent ligand recognition. This indicates that there are multiple binding sites on the α- and β-subunits of LFA-1 that interact with ICAM-1.^19,20^ The activities of cLABL and cLBEL are consistent with the widely published reports on the characteristics of ICAM-1/LFA-1 interaction in which the α-subunit I-domain and the I-like domain of β-subunit are the active components of various integrins for interaction with their ligands.

The lower inhibitory activity of the conjugate compared to its parent peptide was proposed to be due to the steric hindrance imposed by MTX during peptide-ICAM-1 interaction (Figures 2 & 4). The MTX conjugation could alter the peptide structure to a less favorable conformation for binding to ICAM-1; this was reflected in 10% reduction of cell adhesion inhibitory activity when the conjugate was compared to the parent peptide. The relatively low loss in inhibitory activity of conjugate was complemented by the conjugate ability to suppress the production of anti-inflammatory cytokines with lower cell toxicity than MTX alone.

The productions of IL-6 and IL-8 in HCAEC were inhibited by peptides, MTX-peptide conjugates, and MTX (Figure 6). In this case, MTX-peptide conjugate selectively targeted ICAM-1 receptor on HCAEC and the conjugate was endocytosed by ICAM-1 into the cytoplasmic domain to exert MTX activity to suppress cytokine production. The proposed mechanism of MTX in modulating cytokine production has been debated and contradictory; the mechanism can be dependent on the type of cells and cytokine being produced.^50–52^ This may explain the difference in the compound inhibitory profiles of IL-6 and IL-8 production. A relatively higher concentration was required to linearly reduce the IL-8 than IL-6 production; this was due to the nature of HCAEC as the most potent IL-8 producers.^50^ IL-8, but not IL-6, has been suggested as an important angiogenesis mediator and a major contributor for the progression of coronary atherosclerotic disease in human.^53^ Although the order of cytokine inhibitory activities seems to correspond to the relative cytotoxicity, this present study cannot confirm the direct correlation between the two. Therefore, the possible existence of other signaling mechanisms cannot be ruled out. Taken together, the present study suggests that the conjugates are effective inhibitors of inflammatory responses with lower toxicity than that of MTX alone.

The cLABL activity to bind ICAM-1 is not only good for inhibition of T cell adhesion but is also useful for inhibiting costimulatory signal that has function to alter the immune response.^31,54^ The cluster of LFA-1/ICAM-1 interactions involves in the formation of immunological synapse, which is responsible for a positive costimulatory signal to stimulate T cell activation in the inflammatory response. Thus, blocking this costimulatory signal cluster by cLABL suppresses T cell activation.^54^ In autoimmune diseases, this costimulatory signal can propagate the autoreactive immune cells against self-antigens.^31,54^ Recently, linear LABL peptide has been conjugated to antigenic peptides that are associated with autoimmune diseases to yield bifunctional peptide inhibitor (BPI) molecules.^31,54^ These BPI molecules can simultaneously be connected to MHC-II and ICAM-1 on the surface of antigen-presenting cells (APC) (i.e., dendritic and B cells) during T cell-APC interaction to inhibit differentiation of naïve T cells to inflammatory T cells (i.e., Th_1_ and Th_17_ cells).^12,31,54^

Various forms of BPI molecules were designed by combining LABL peptide and different antigenic peptides that are derived from myelin sheath proteins of the axon, including myelin basic protein (MBP), myelin oligodendrocyte glycoprotein (MOG), and proteolipid protein (PLP).^31^ PLP-BPI and MOG-BPI are conjugates between PLP or MOG peptide with LABL peptide, respectively. BPI molecules suppressed various mouse models of the experimental autoimmune encephalomyelitis (EAE) disease as the animal model of MS.^35,36^ Similarly, the GAD-BPI molecule containing LABL and antigenic peptide from glutamic acid decarboxylase 65 kDa (GAD65) suppressed type-1 diabetes (T1D) in the mouse model.^37^ In addition, CII-BPI molecules that are conjugates between LABL and collagen-II peptides suppressed rheumatoid arthritis (RA) in collagen-induce arthritis (CIA) mice.^38^ The concept of BPI was extended to protein and polymer conjugates such as Fc-BPI, I-domain antigen conjugate (IDAC), and soluble antigen array (SAgA) molecules; they are composed of LABL and antigenic peptides.^31^ These large conjugates have been shown to effectively suppress EAE in the mouse model. Most BPI molecules suppress inflammatory Th_1_ and Th_17_ immune cells and stimulate regulatory immune cells (T_reg_ cells).

## CONCLUSION

The MTX-cLABL and MTX-cLBEL conjugates targeted ICAM-1 on HCAEC cells and they also inhibited IL-6 and IL-8 production as inflammatory cytokines with lower toxicity compared to MTX. These conjugates can suppress the inflammatory immune response via dual mechanisms. First, the conjugates inhibited LFA-1/ICAM-1 interactions as a costimulatory signal as well as T cell adhesion to HCAEC to prevent T cell activation. Secondly, the conjugate goes through ICAM-1-mediated endocytosis to delivery MTX into the cytoplasmic domain of HCAEC for suppressing IL-6 and IL-8 production. In summary, these peptides can also be used to target other drugs to ICAM-1-expressing cells for lowering drug side effects and controlling the inflammatory immune response.

## Figures and Tables

**Scheme 1:**